# Validation of the advanced‐stage Hodgkin lymphoma international prognostic index (A‐HIPI) in the GHSG HD21 trial

**DOI:** 10.1002/hem3.70471

**Published:** 2026-09-11

**Authors:** Hishan Tharmaseelan, Janina Jablonski, Susan K. Parsons, Angie Mae Rodday, Andrew M. Evens, Josée M. Zijlstra, Alexander Fosså, Daniel Molin, Mark Hertzberg, Felicitas Hitz, Martin Fehr, Richard Greil, Thomas Melchardt, Michael Fuchs, Helen Kaul, Peter Borchmann, Justin Ferdinandus

**Affiliations:** ^1^ Faculty of Medicine and University Hospital of Cologne, Department I of Internal Medicine, Center for Integrated Oncology Aachen Bonn Cologne Düsseldorf (CIO ABCD), and German Hodgkin Study Group (GHSG) University of Cologne Cologne Germany; ^2^ Division of Hematology/Oncology and Institute for Clinical Research and Health Policy Studies Tufts Medical Center Boston Massachusetts United States of America; ^3^ Division of Blood Disorders Rutgers Cancer Institute New Jersey New Brunswick New Jersey United States of America; ^4^ Department of Hematology Amsterdam UMC, Vrije Universiteit, Cancer Center Amsterdam Amsterdam The Netherlands; ^5^ Department of Oncology Oslo University Hospital Oslo Norway; ^6^ Department of Immunology, Genetics and Pathology Uppsala University Uppsala Sweden; ^7^ Cancer Immunotherapy Uppsala University Uppsala Sweden; ^8^ Department of Haematology Prince of Wales Hospital Sydney New South Wales Australia; ^9^ Australasian Leukaemia Lymphoma Group (ALLG) Melbourne Victoria Australia; ^10^ Swiss Cancer Institute Competence Center Bern Switzerland; ^11^ Department of Medical Oncology and Haematology Cantonal Hospital St. Gallen St. Gallen Switzerland; ^12^ Austrian Group for Medical Tumor Therapy (AGMT) Salzburg Austria; ^13^ Salzburg Cancer Research Institute – Center for Clinical Cancer and Immunology Trials (SCRI‐CCCIT) Salzburg Austria; ^14^ Department of Internal Medicine III With Haematology, Medical Oncology, Haemostaseology, Infectiology and Rheumatology, Cancer Research Laboratory of the Department of Internal Medicine III Paracelsus Medical University Salzburg Austria

The International Prognostic Score (IPS) has served as the standard risk stratification tool in advanced‐stage classical Hodgkin Lymphoma (cHL) for over two decades.^1^ The IPS‐7 incorporates seven dichotomized clinical factors—age, sex, serum albumin, hemoglobin, Ann Arbor stage, leukocytosis, and lymphocytopenia—to assign patients into risk categories for disease progression at 5 years. The score's ability for prognostication has diminished considerably as modern regimens have led to substantially improved outcomes.^2^, ^3^

The advanced‐stage Hodgkin lymphoma international prognostic index (A‐HIPI) was developed with contemporary statistical methods as a prognostic model using individual patient data pooled from eight clinical trials and was validated in prominent registries.^4^ A‐HIPI models employ, with slight variation, age, lymphocyte count, hemoglobin, and albumin levels as continuous variables besides sex, Ann Arbor stage, and bulky disease to predict 5‐year (5y) probabilities of progression‐free survival (PFS) and overall survival (OS). A‐HIPI demonstrated improved calibration for PFS and OS and enhanced discrimination for OS compared to IPS‐7. The original model was developed and validated primarily with ABVD‐treated patients, while additional validations have been published.^5^, ^6^, ^7^ There is a need to validate prediction models for patients treated with novel agents such as brentuximab vedotin (Bv)^8^ or nivolumab,^9^, ^10^ which are increasingly used in the first‐line treatment.^11^

The randomized, Phase III GHSG HD21 trial (NCT02661503) compared BrECADD (brentuximab vedotin, etoposide, cyclophosphamide, doxorubicin, dacarbazine, and dexamethasone) with eBEACOPP (escalated bleomycin, etoposide, doxorubicin, cyclophosphamide, vincristine, procarbazine, and prednisone) in advanced‐stage cHL.^12^ The trial demonstrated superior efficacy of BrECADD compared to eBEACOPP. The aim of this analysis was to assess the performance of the A‐HIPI in the HD21 trial population and to compare it with the IPS‐7. This analysis included 1446 patients from HD21 (BrECADD: 729; eBEACOPP: 717). The cohort was restricted to intention‐to‐treat (ITT) patients with available A‐HIPI variables and laboratory values within the predefined ranges (S1).

The full model equations of the A‐HIPI have been reported elsewhere.^4^ To assess the model's performance, discrimination and calibration were used. Calibration measures the model's prediction accuracy and was assessed by comparing observed and predicted probabilities of 5y‐events within quintiles of predicted risk. For observed event rates, the Kaplan–Meier method was used. Calibration plots were created using local regression (loess) and intercepts and slopes were estimated using linear regression. A perfectly calibrated model would have an intercept of 0 and a slope of 1. Calibration‐in‐the‐large was calculated by subtracting overall observed event rates from predicted ones. Kaplan–Meier curves were generated by quintiles of predicted risk for events. Discrimination, defined as the model's ability to rank patients according to their actual risk, was compared between A‐HIPI and IPS‐7 using Harrell's *C*‐statistic and its difference, with 95% bootstrap confidence intervals derived from 2000 iterations. Analyses were performed for the total cohort and separately for each treatment arm.

Patient and disease characteristics of the HD21 cohort have been described elsewhere.^12^ Of the 1473 patients in the HD21 ITT population, 1446 (98.2%) were evaluable for the A‐HIPI analysis (S2). Exclusions occurred due to missing clinical stage (*n* = 4) and out of range laboratory values (*n* = 23). The distributions of A‐HIPI‐predicted probabilities for both PFS and OS were comparable between the HD21 validation and the original development cohort, indicating that the patient populations were similar in their risk profiles.

In the HD21 validation cohort, 27 OS events and 112 PFS events occurred, resulting in a 5y‐OS of 98.2% (95% CI 97.5–98.9) and 5y‐PFS of 92.3% (95% CI 90.9–93.7) compared to a 5y‐OS of 92% (95% CI 91–93) and 5y‐PFS of 77% (95% CI 76–78) in the original development cohort. Therefore, calibration‐in‐the‐large revealed substantial overestimation of 5y‐PFS events by A‐HIPI (23.9% predicted vs. 7.7% observed event rate), yielding a discrepancy of 16.2 percentage points. The calibration intercept was 0.0, and the slope was 0.32 (95% CI 0.07–0.57), markedly below the ideal value of 1, indicating poor calibration in risk quintiles (Figure 1). The difference between A‐HIPI‐predicted and observed event rates increased progressively from quintile 0 (11.7 percentage points) to quintile 4 (22.7 percentage points), demonstrating that the overestimation was most pronounced in patients classified as high risk. Regarding discrimination, the A‐HIPI *C*‐statistic was 0.57 (95% CI 0.52–0.63), compared to 0.61 (95% CI 0.56–0.66) for the IPS‐7 with a difference of −0.04 (95% CI −0.08–0.01), indicating modest discriminative ability for both models and a slight advantage of the IPS‐7. Kaplan–Meier analysis by quintiles of event probabilities predicted by A‐HIPI in the HD21 cohort demonstrated that, in concordance with overall modest discrimination, the model showed poor ability to stratify patients in terms of PFS (S3). Cumulative incidence plots and 3‐ and 5‐year PFS estimates by quintile are provided in S4/S5. The limited number of OS events precluded a meaningful assessment of the survival model.

**Figure 1:**
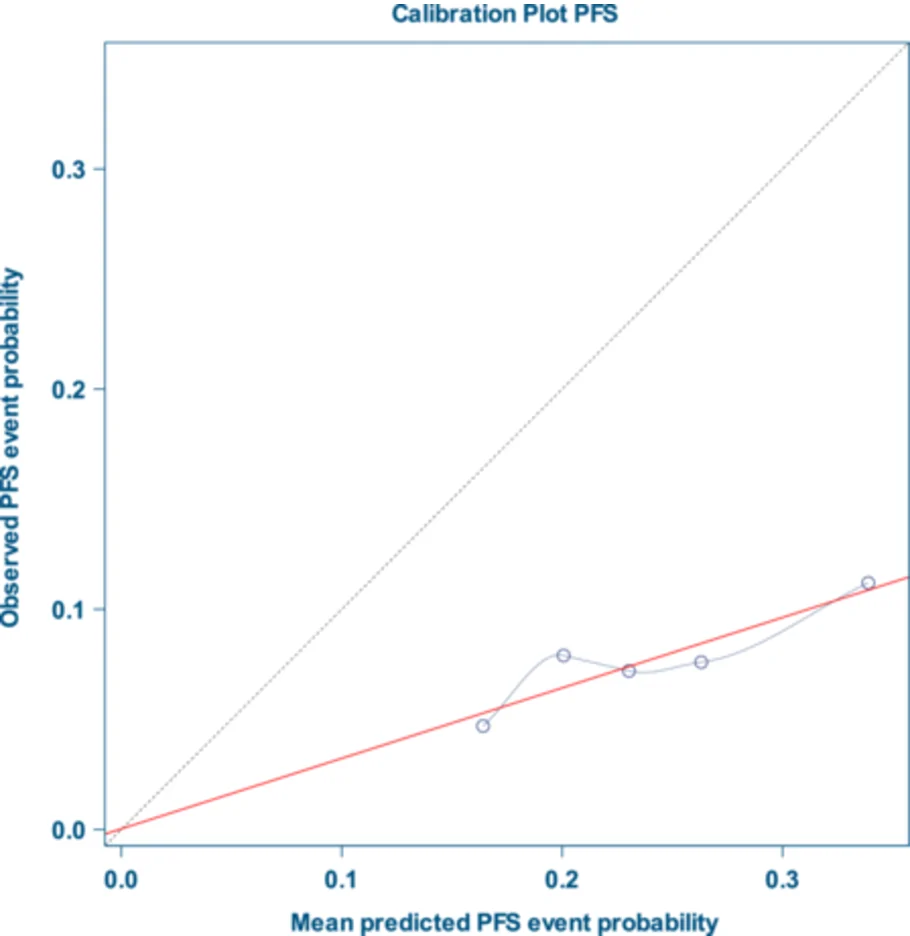
Calibration plot of observed versus advanced‐stage Hodgkin lymphoma international prognostic index (A‐HIPI)‐predicted 5‐year progression‐free survival (PFS) event probabilities by quintiles in the HD21 validation cohort, with calibration intercept and slope.

Split by treatment arm, among the 729 patients treated with BrECADD, the A‐HIPI demonstrated a *C*‐statistic for PFS events of 0.61 (95% CI 0.52–0.69) compared to 0.64 (95% CI 0.55–0.73) for the IPS‐7. A notable finding was that patients in the quintile with the lowest risk for PFS events showed a better outcome compared to the remaining quintiles, suggesting that the A‐HIPI may retain clinical utility in identifying a low‐risk subgroup within this treatment arm (Figure 2A). The calibration‐in‐the‐large indicated an 18‐percentage point overestimation of events, the calibration intercept was −0.02, and the slope was 0.33 (95% CI −0.03 to 0.68).

**Figure 2:**
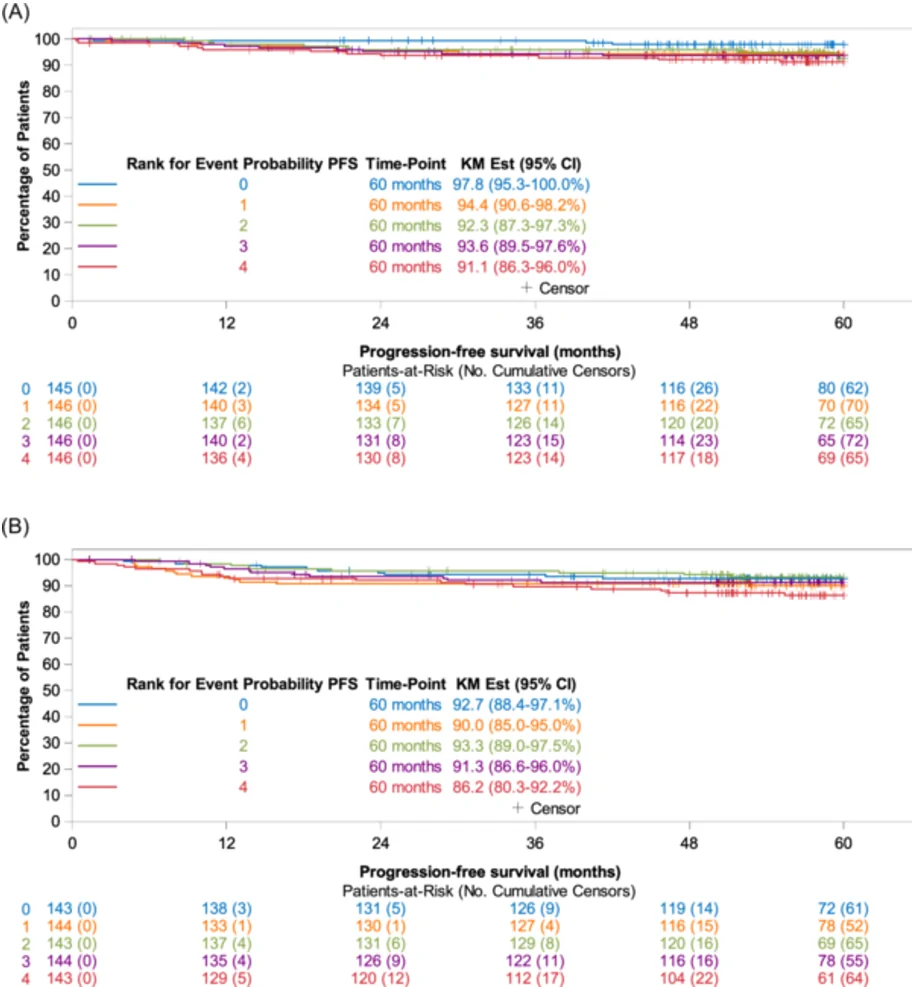
**Kaplan–Meier plots of progression‐free survival by advanced‐stage Hodgkin lymphoma international prognostic index (A‐HIPI) risk quintiles stratified by treatment arm: (A) brentuximab vedotin, etoposide, cyclophosphamide, doxorubicin, dacarbazine, and dexamethasone (BrECADD) and (B) escalated bleomycin, etoposide, doxorubicin, cyclophosphamide, vincristine, procarbazine, and prednisone (eBEACOPP).** PFS, progression‐free survival.

In the 717 patients receiving eBEACOPP, the A‐HIPI *C*‐statistic for PFS events was 0.55 (95% CI 0.49–0.63), while the IPS‐7 achieved 0.59 (95% CI 0.52–0.65). Both indices showed numerically lower discrimination in the eBEACOPP compared to the BrECADD arm. In this arm, the quintile with the highest risk for PFS events demonstrated a worse outcome compared to the other quintiles (Figure 2B). Calibration‐in‐the‐large indicated a 15‐percentage point overestimation of events—somewhat less than in the BrECADD arm, likely reflecting the higher absolute event rate in this treatment group. The calibration intercept was 0.01, and the slope was 0.33 (95% CI −0.17 to 0.83).

This analysis presents the first evaluation of A‐HIPI in a large trial cohort treated with BrECADD. While the distribution of A‐HIPI scores in HD21 patients mirrored that of the development cohort, the model substantially overestimated event rates for PFS; probably due to the baseline survival underlying the model being too low relative to outcomes achieved with modern therapies. Additionally, calibration slopes of approximately 0.32–0.33 across all analyses indicate the model's poor calibration in risk subgroups of the HD21 population. Consequently, recalibrating baseline survival may improve calibration‐in‐the‐large but would neither affect the calibration slope nor the model's discrimination.

Despite calibration limitations, A‐HIPI retained partial discriminative value in both treatment arms, though in different directions. In the BrECADD arm, the lowest risk quintile separated well from the rest, suggesting potential clinical utility for identifying patients who might be candidates for treatment de‐escalation. Conversely, in the eBEACOPP arm, the highest risk quintile was most informative, identifying patients with relatively worse outcomes who may warrant closer monitoring or alternative strategies. This pattern of differential discrimination across treatment arms warrants further investigation and might indicate that the A‐HIPI does not function independent from treatment.

In a machine‐learning–based approach by Jørgensen et al., approximately 25% of patients had received BEACOPP, yielding prognostic improvement over IPS, but very similar prognostic performance to A‐HIPI.^13^ Other analyses support the discriminatory power of A‐HIPI with superior performance to IPS. For instance, in a Turkish real‐world cohort by Koca et al., almost all patients (202 of 207 total, 97.6%) had received ABVD‐based treatment, leading to the—perhaps premature—conclusion that the IPS is outdated.^5^ Another validation in the Brazilian Hodgkin lymphoma registry showed similar superior prognostic power for the A‐HIPI with 92% of patients having been treated with ABVD.^6^ In the only analysis of A‐HIPI incorporating novel agents, the evaluation in Bv‐/Nivolumab‐AVD treated patients by Casulo et al., *C*‐statistics were improved compared to IPS (A‐HIPI: 0.63 [95% CI 0.58–0.68]; IPS‐7: 0.59 [95% CI 0.54–0.65]).^7^ These observations suggest that the A‐HIPI remains valid in patients treated with less effective, ABVD‐based regimens, which are still used throughout the world.

In the current analysis, IPS‐7 consistently showed numerically higher *C*‐statistics than the A‐HIPI, though confidence intervals of the difference exceeded 0. This may reflect the fact that both models operate in a population with very low event rates. Importantly, neither model demonstrated strong discriminative ability, underscoring the challenge of risk stratification in an era of highly effective therapies.

Several limitations merit acknowledgment. Low event rates limited statistical power, particularly for OS analyses. The analysis was restricted to trial participants, who may not fully represent real‐world populations.

In conclusion, while the A‐HIPI model retains partial utility in identifying extreme risk groups within treatment arms, systematic overestimation of event rates and absence of a performance advantage compared to IPS‐7 strongly limit its prognostic validity for patients treated with highly effective regimens. Circulating tumor DNA^14^ and serum TARC,^15^ both as baseline and dynamic biomarkers, represent promising prognostic markers that could refine treatment stratification. As frontline therapy becomes increasingly effective, the identification of novel markers is warranted to develop baseline‐ and on‐treatment risk‐adapted treatment strategies.

## AUTHOR CONTRIBUTIONS

**Hishan Tharmaseelan**: Conceptualization; methodology; writing—original draft; writing—review and editing. **Janina Jablonski**: Conceptualization; formal analysis; data curation; methodology; writing—review and editing. **Susan K. Parsons**: Writing—review and editing. **Angie Mae Rodday**: Writing—review and editing. **Andrew M. Evens**: Writing—review and editing. **Josée M. Zijlstra**: Writing—review and editing. **Alexander Fossa**: Writing—review and editing. **Daniel Molin**: Writing—review and editing. **Mark Hertzberg**: Writing—review and editing. **Felicitas Hitz**: Writing—review and editing. **Martin Fehr**: Writing—review and editing. **Richard Greil**: Writing—review and editing. **Thomas Melchardt**: Writing—review and editing. **Michael Fuchs**: Writing—review and editing. **Helen Kaul**: Writing—review and editing. **Peter Borchmann**: Supervision; writing—review and editing. **Justin Ferdinandus**: Conceptualization; supervision; writing—review and editing.

## CONFLICT OF INTEREST STATEMENT

T.M.—honoraria: Takeda. D.M.—honoraria: Roche. R.G.—advisory role: Celgene, Novartis, Roche, BMS, Takeda, AbbVie, AstraZeneca, Janssen, MSD, Merck, Gilead, and Daiichi Sankyo; stock ownership: Novo Nordisk, Lilly, Regeneron, and Vertex; honoraria: Celgene, Roche, Merck, Takeda, AstraZeneca, Novartis, Amgen, BMS, MSD, Sandoz, AbbVie, Gilead, Daiichi Sankyo, Sanofi, and Pfizer; research funding: Celgene, Merck, Takeda, AstraZeneca, Novartis, Amgen, BMS, MSD, Sandoz, Gilead, and Roche; other (e.g., travel support): Roche, Amgen, AstraZeneca, Janssen, Novartis, MSD, Celgene, Gilead, BMS, Abbvie, and Daiichi Sankyo. M.H.—advisory/honoraria: Takeda, Roche, AstraZeneca, and Specialised Therapeutics. Ma.F.—travel support: Takeda. P.B.—advisory role: Takeda Oncology, BMS, Roche, Miltenyi Biomedicine, Kite‐Gilead, MSD, and Incyte; honoraria: Takeda Oncology, BMS, Roche, MSD, Miltenyi Biomedicine, Kite‐Gilead, Sobi, and AbbVie; research funding: Takeda Oncology, MSD, and Incyte.

## ETHICS STATEMENT

Ethics approval was granted by the Ethics Committee of the Medical Faculty at the University of Cologne (reference number 16‐008). All patients provided written informed consent before study entry according to the Good Clinical Practice guidelines of the International Council for Harmonisation of Technical Requirements for Pharmaceuticals for Human Use.

## FUNDING

This research received no funding. Open Access funding enabled and organized by Projekt DEAL.

## Supporting information

Supporting Information.

## Data Availability

Individual patient data from the GHSG HD21 trial (NCT02661503) may be made available upon reasonable request to the German Hodgkin Study Group, subject to approval by relevant data access committees. Requests should be directed to the corresponding author.
