# Supplementary material for: Validation of the advanced‐stage Hodgkin lymphoma international prognostic index (A‐HIPI) in the GHSG HD21 trial
Source: Hemasphere. 2026 Sep 11;10(9):e70471. doi: 10.1002/hem3.70471 (PMC13567801; doi:10.1002/hem3.70471)
Supplement: Supplementary file 1 [file HEM3-10-e70471-s001.docx]

**Supplements**

**S1: Defined value ranges according to A-HIPI original publication**

Age: 18-65; Albumin: 1-6 g/dL; Hemoglobin: 5-16.5 g/dL; Lymphocyte count: 0.1 – 5.0 10^3^ /µl

**S2: Consort flow diagram**

**S3: PFS by risk quintiles**

# Kaplan-Meier plots of progression-free survival by A-HIPI risk quintiles in the HD21 total cohort. Quintile 0 represents the lowest predicted risk and quintile 4 the highest predicted risk.

**S4: Cumulative incidence plots for all patients and split by arm**

Cumulative incidence over all patients

Cumulative incidence for BrECADD patients

Cumulative incidence for eBEACOPP patients

**S5: Quintile rank progression free survival 3-year and 5-year estimates**

|  | **Total** | | **BrECADD** | | **eBEACOPP** | |
| --- | --- | --- | --- | --- | --- | --- |
| **Rank for Event Probability PFS** | 3y-PFS est.  % (95% CI) | 5y-PFS est.  % (95% CI) | 3y-PFS est.  % (95% CI) | 5y-PFS est.  % (95% CI) | 3y-PFS est.  % (95% CI) | 5y-PFS est.  % (95% CI) |
| **0** | 96.8  (94.8- 98.9) | 95.3  (92.9-97.8) | 99.3  (97.9-100) | 97.8  (95.3-100) | 94.3  (90.4-98.1) | 92.7  (88.4-97.1) |
| **1** | 92.6  (89.6- 95.7) | 92.1  (89.0-95.3) | 94.4  (90.6-98.2) | 94.4  (90.6-98.2) | 90.9  (86.2-95.6) | 90.0  (85.0-95.0) |
| **2** | 95.7  (93.3- 98.1) | 92.8  (89.5-96.1) | 95.7  (92.3-99.1) | 92.3  (87.3-97.3) | 95.7  (92.3-99.1) | 93.3  (89.0-97.5) |
| **3** | 93.2  (90.2-96.2) | 92.4  (89.3-95.5) | 94.4  (90.5-98.2) | 93.6  (89.5-97.6) | 92.1  (87.6-96.6) | 91.3  (86.6-96.0) |
| **4** | 91.7  (88.5-95.0) | 88.8  (84.9-92.6) | 93.6  (89.6-97.7) | 91.1  (86.3-96.0) | 89.7  (84.6-94.8) | 86.2  (80.3-92.2) |
